# GWA Mapping of Anthocyanin Accumulation Reveals Balancing Selection of *MYB90* in *Arabidopsis thaliana*

**DOI:** 10.1371/journal.pone.0143212

**Published:** 2015-11-20

**Authors:** Johanna A. Bac-Molenaar, Emilie F. Fradin, Juriaan A. Rienstra, Dick Vreugdenhil, Joost J. B. Keurentjes

**Affiliations:** 1 Laboratory of Plant Physiology, Wageningen University, Wageningen, The Netherlands; 2 Laboratory of Genetics, Wageningen University, Wageningen, The Netherlands; NARO Institute of Fruit Tree Science, JAPAN

## Abstract

Induction of anthocyanin accumulation by osmotic stress was assessed in 360 accessions of *Arabidopsis thaliana*. A wide range of natural variation, with phenotypes ranging from green to completely red/purple rosettes, was observed. A genome wide association (GWA) mapping approach revealed that sequence diversity in a small 15 kb region on chromosome 1 explained 40% of the variation observed. Sequence and expression analyses of alleles of the candidate gene *MYB90* identified a causal polymorphism at amino acid (AA) position 210 of this transcription factor of the anthocyanin biosynthesis pathway. This amino acid discriminates the two most frequent alleles of *MYB90*. Both alleles are present in a substantial part of the population, suggesting balancing selection between these two alleles. Analysis of the geographical origin of the studied accessions suggests that the macro climate is not the driving force behind positive or negative selection for anthocyanin accumulation. An important role for local climatic conditions is, therefore, suggested. This study emphasizes that GWA mapping is a powerful approach to identify alleles that are under balancing selection pressure in nature.

## Introduction

Accumulation of anthocyanins in the vacuole of plants is the most clearly visible sign of stress. Anthocyanins have been studied for decades because of their strong color that directly attracts the eye of the observer. They are best known from their appearance during the ripening of fruits [1] and from their accumulation in autumn leaves [2], but they also play a prominent role in many stress responses. Accumulation of anthocyanins upon stress exposure is a conserved and desirable trait. It is found in many plant species upon exposure to a wide variety of biotic and abiotic stresses [3–7]. Anthocyanins protect plants against stress owing to four properties. First, anthocyanins protect the photosynthetic machinery of plants exposed to an excess of light (especially UV-B) by absorbing blue light and reflecting red light [8]. Secondly, anthocyanins possess anti-oxidant properties, which may scavenge reactive compounds known to occur under many stresses, such as reactive oxygen species, and, therefore, increase a plant's tolerance towards stress [9]. Thirdly, anthocyanins may bind to various heavy metals, providing a protective role in metal-tolerance by sequestering these toxic elements [10]. Finally, anthocyanins are assumed to exhibit an osmo-regulatory function, allowing a plant to remain turgid under low water availability [11, 12]. Because of these characteristics, induced anthocyanin accumulation is a desirable trait in many environments in which plants are temporarily experiencing stresses.

Anthocyanins are the end products of a specific branch of the flavonoid biosynthesis pathway (S1 Fig), (for a review of this pathway see [13, 14]). The expression of the structural and regulatory genes of the anthocyanin pathway have been studied in detail in the *Arabidopsis thaliana* accession *Col-0* and for each step in the pathway at least one mutant in the *Col-0* background has been studied [15]. These analyses revealed that many structural genes in the pathway are essential for the synthesis of anthocyanins but that functional redundancy exists for transcriptional regulators (S1 Fig). Biosynthesis genes early in the pathway are regulated differently than the genes at the end of the pathway [16]. Early biosynthesis genes, leading to the production of flavonols, are regulated by R2R3-MYB transcription factors (*MYB11*, *MYB12* and *MYB111*). The biosynthesis genes later in the pathway, leading to the synthesis of pro-anthocyanidins and anthocyanins, are regulated by a complex of MYB, bHLH and WD40 transcription factor families [17]. *MYB75* (*PRODUCTION OF ANTHOCYANIN PIGMENT 1*, *PAP1*), *MYB90* (*PRODUCTION OF ANTHOCYANIN PIGMENT 2*, *PAP2*), *MYB113* and *MYB114* display redundant functions and can all be part of this complex [16]. Overexpression studies of these four MYB transcription factors revealed that the up-regulation of either one of them is sufficient to increase anthocyanin accumulation in young leaves and upon osmotic stress [16, 18]. In addition to these transcription factors, many other structural and regulatory genes of the pathway are up-regulated in the reference accession Col-0 under various stress conditions [4, 19–21]. Moreover, a few studies report on variation in constitutive or stress induced anthocyanin accumulation in different accessions of Arabidopsis [5, 12, 22, 23]. For *MYB75* (*PAP1*) and *MYB90 (PAP2)*, sequence variation has been reported to be causal for natural variation in anthocyanin accumulation [24–26]. Although these studies identified natural variation in the ability to accumulate anthocyanins and even detected causal genes explaining the observed variation, little insight was gained on their role in natural populations. Genetic analyses of recombinant inbred lines (RILs), for instance, provides no information about the prevalence of allelic differences or on the selective forces that may shape the genetic architecture of populations.

Sequence diversity that causes differences in anthocyanin accumulation will be subject to natural selection. Depending on the environment, low or high constitutive anthocyanins levels and strong or moderate induction of anthocyanin production will be favored. Here we investigated natural variation in constitutive and induced anthocyanin accumulation in 360 natural accessions of Arabidopsis. GWA mapping revealed the transcription factor *PAP2* (*MYB90*) as the major gene responsible for the observed variation in induced anthocyanin accumulation. Two alleles of this gene dominate the world-wide population, suggesting balancing selection for the polymorphism discriminating these two alleles. No link could be found between the origin of collection of the accessions and the amount of constitutive or induced anthocyanin accumulation. These results suggest that not the macro climate, but the local climate conditions might be the driving force behind positive or negative selection for anthocyanin accumulation.

## Results

Accumulation of anthocyanins in the leaves can be favorable for a plant because of their light-shielding, metal-binding and antioxidant capacity, and their function in osmotic-regulation. However, this process is also energy-demanding and, therefore, not favorable under all conditions. In this study we investigated the natural variation in constitutive and induced anthocyanin accumulation and used a GWA mapping approach to search for regulatory genes that are underlying the natural variation in anthocyanin accumulation.

### Anthocyanin accumulation is genotype dependent

**Fig 1:**
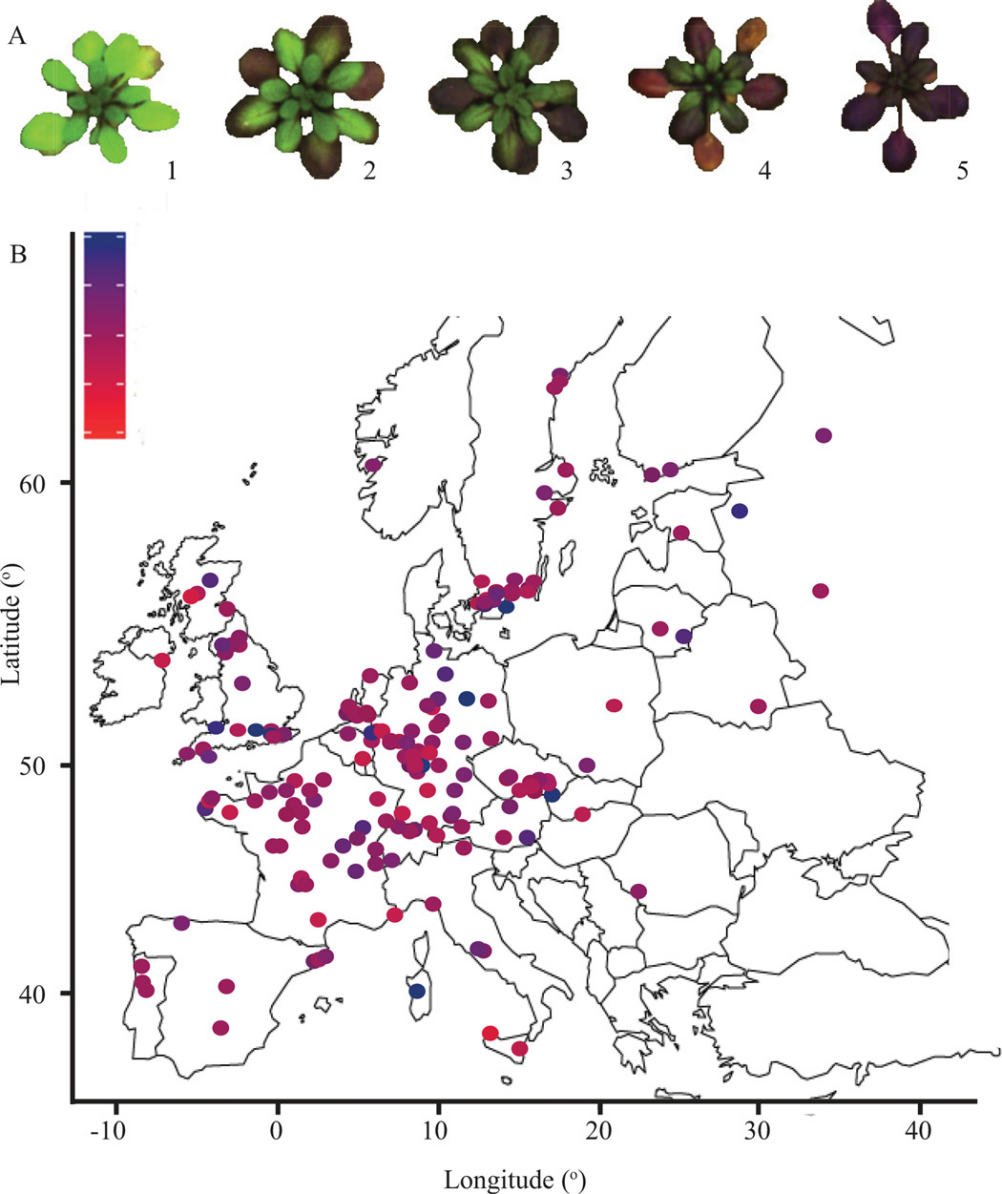
Natural variation in anthocyanin accumulation. **A)** Representative examples of categories of anthocyanin accumulation. Anthocyanin accumulation was scored visually by assigning each individual plant to an accumulation category. **B)** Geographical distribution of the accessions in Europe. The color indicates the anthocyanin accumulation category in stress conditions.

**Fig 2:**
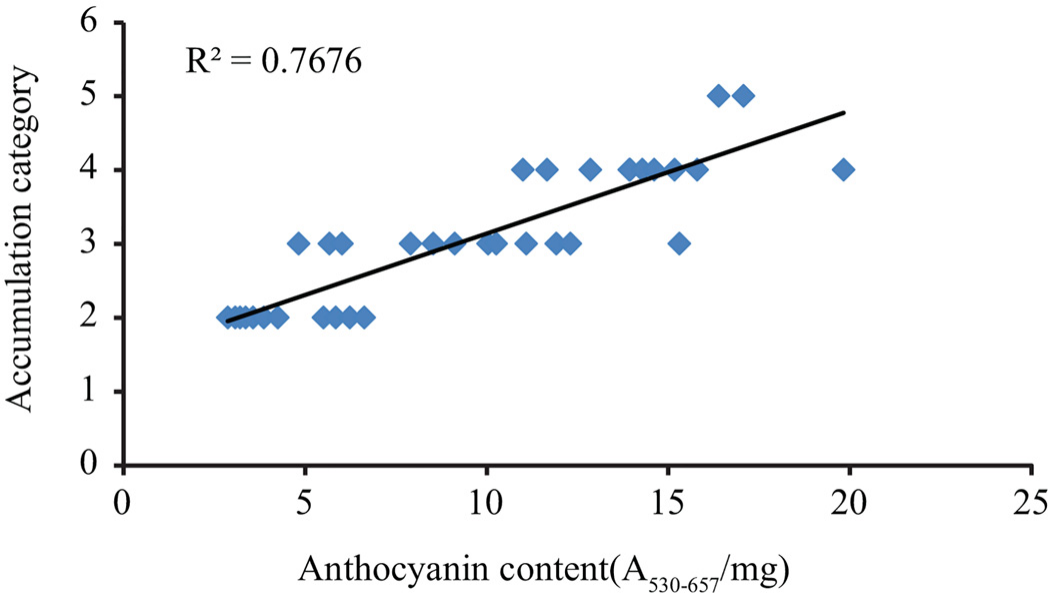
Linear correlation between accumulation category assigned to plants by visual scoring and anthocyanin content quantified spectrophotometrically.

**Fig 3:**
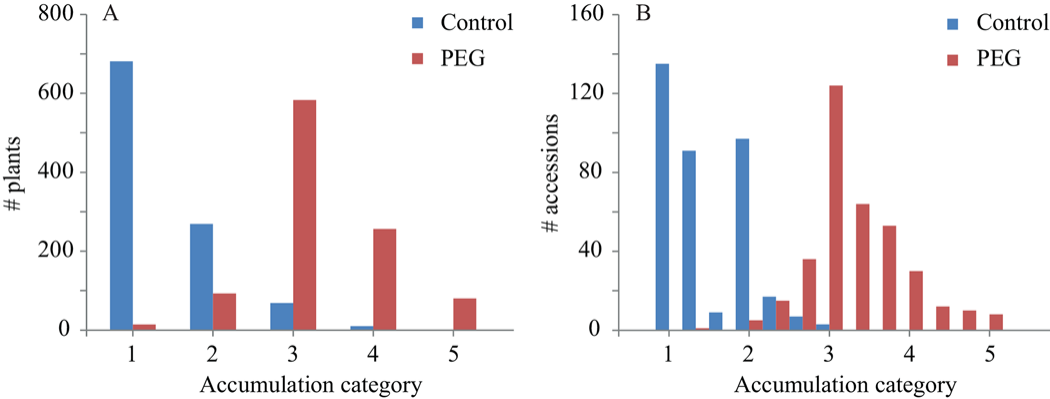
Frequency distribution over the scoring categories. In total 2160 plants were scored, three replicates of each of the 360 accessions in control and in PEG treated conditions. **A)** Frequency distribution based on number of plants. **B)** Frequency distribution based on number of accessions.

Anthocyanin accumulation for 360 natural accessions of Arabidopsis was evaluated by visual scoring of rosette-stage plants grown under control conditions or osmotic stress conditions. Each plant was assigned to a category ranging from a completely green plant (category 1) to a completely red/purple plant (category 5) (Fig 1A). These categories correlated very well with anthocyanin content as determined by spectrophotometric analyses (Fig 2). Most plants (66%) did not produce any visible anthocyanins under control conditions and only very few accumulated substantial levels (Fig 3). PEG treatment, however, induced anthocyanin accumulation in almost all accessions, with most plants being assigned to category 3 and more than 98% to category 2 or higher (Fig 3A). Levels of accumulation were highly reproducible among replicates of accessions within and between experiments. Illustratively, a high correlation was observed between anthocyanin accumulation in the association mapping experiment and the confirmation experiment (Spearman’s ρ = 0.93, p = 0.04*10^−11^). The broad sense heritability of anthocyanin accumulation under control conditions was 35% and 47% under stress conditions, indicating moderate to strong genetic control. A weak, but significant correlation was detected between anthocyanin accumulation under control and treatment conditions (Spearman’s ρ = 0.19, p = 0.00033), indicating that anthocyanin formation depends partly on the genetic background, independent of the environment. One accession (Pa-1, CS76204) responded to the osmotic stress with signs of early senescence. The leaves turned yellow without detectable levels of anthocyanin.

### Evolution of anthocyanin accumulation is independent of macro-climatic differences

To identify geographical clines in the ability to accumulate high levels of anthocyanins, the origin of collection of all accessions tested was analysed. Most of the accessions (321 out of 360) originate from Europe. To avoid bias due to low observation numbers in other continents, only the European accessions were used to identify patterns in the distribution of the different accumulation categories. Correlations between anthocyanin accumulation and latitude and longitude were calculated. A weak significant correlation was found between the latitude and the accumulation of anthocyanins under control (ρ = 0.15, p = 0.006) and stress (ρ = 0.13, p = 0.023) conditions. This suggests a trend of northern accessions accumulating more anthocyanin than southern accessions (Fig 1B). However, as can be expected from the weak clines, accessions with different accumulation phenotypes often occur in close range of each other. In the United Kingdom, for example, accessions of the whole range of accumulation under both control and stress conditions occur, indicating that macro climate is not the most important factor determining selection of constitutive and stress-induced anthocyanin accumulation.

### GWA mapping reveals a strong association on chromosome 1

**Fig 4:**
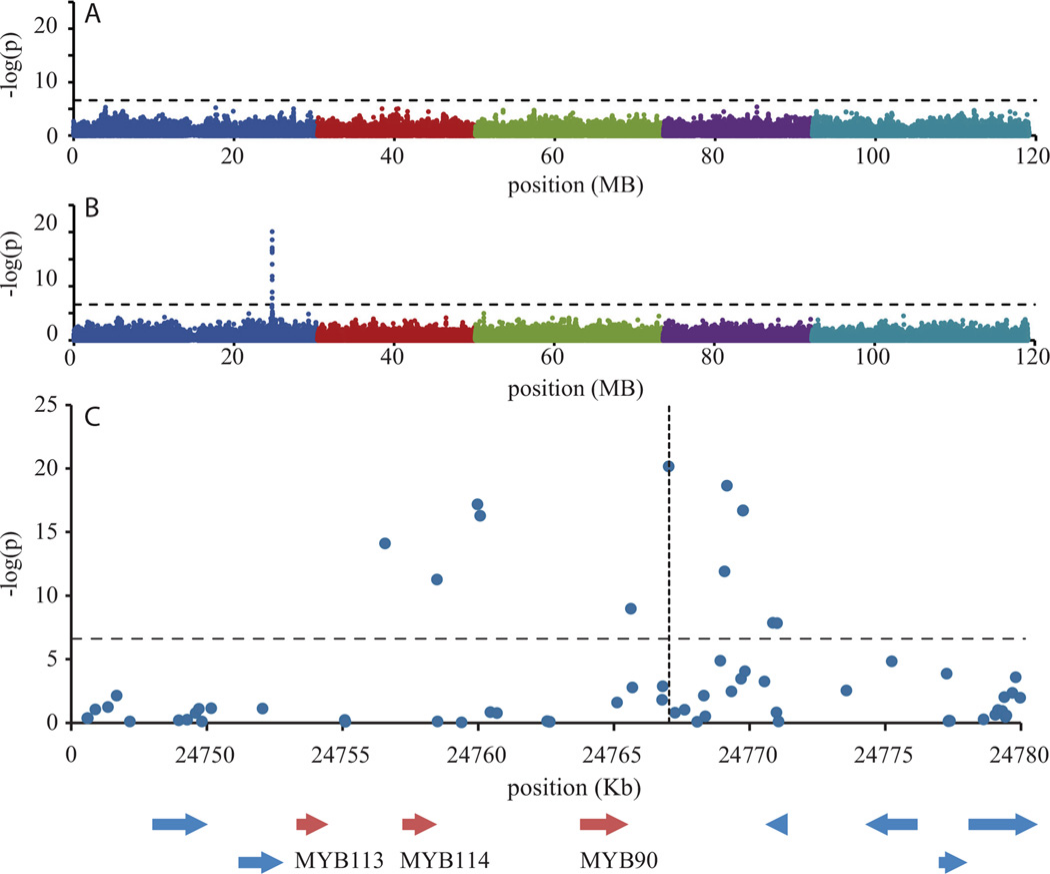
Manhattan plots of GWA mapping for anthocyanin accumulation under control (A) and stress conditions (B). A Bonferroni-corrected threshold of α = 0.05 is represented by the dashed line. Each dot represents a SNP and different colors indicate the five chromosomes of Arabidopsis. **(C)** Blow-up of the region around the significantly associated SNPs. All genes positioned in the region are represented by arrows (red color: genes known to play a role in anthocyanin accumulation).

In order to detect causal loci for the variation in anthocyanin accumulation, GWA mapping was applied. This resulted in the detection of a highly significant association of 12 SNPs with the accumulation of anthocyanins induced by PEG (Fig 4B and S2 Table). This locus spans a region of 15 kb on chromosome 1 (Fig 4C). Three genes are located in this region, viz., a calmodulin-like gene (AT1G66400) and two transcription factors of the MYB-family, *MYB114* (AT1G66380) and *MYB90* (*PAP2*, AT1G66390). Both transcription factors have been annotated to play a role in anthocyanin accumulation [16]. A third transcription factor (*MYB113* (AT1G66370), also annotated to play a role in anthocyanin biosynthesis [16], is located just at the border of the associated region. The most significant SNP (–log(p-value) = 20.19) explained 43% of the variance and contributed an effect size of 0.91. The latter indicates that plants of the Columbia haplotype were on average categorized almost one class higher than plants of the non-Columbia haplotype. Genome wide association mapping of anthocyanin accumulation under control conditions did not lead to any association above the Bonferroni corrected significance threshold (Fig 4A), indicating that the chromosome 1 locus does not play a major role in constitutive anthocyanin accumulation. To discover whether the strong association on chromosome 1 masked other weak associations, the most significant SNP (chr1, position 24769177) was used as a cofactor in the mixed model analysis [27, 28]. The conditional GWA mapping, however, did not result in additional SNPs above the Bonferroni corrected threshold.

### Anthocyanin accumulation is not determined by the level of gene expression of MYBs

**Fig 5:**
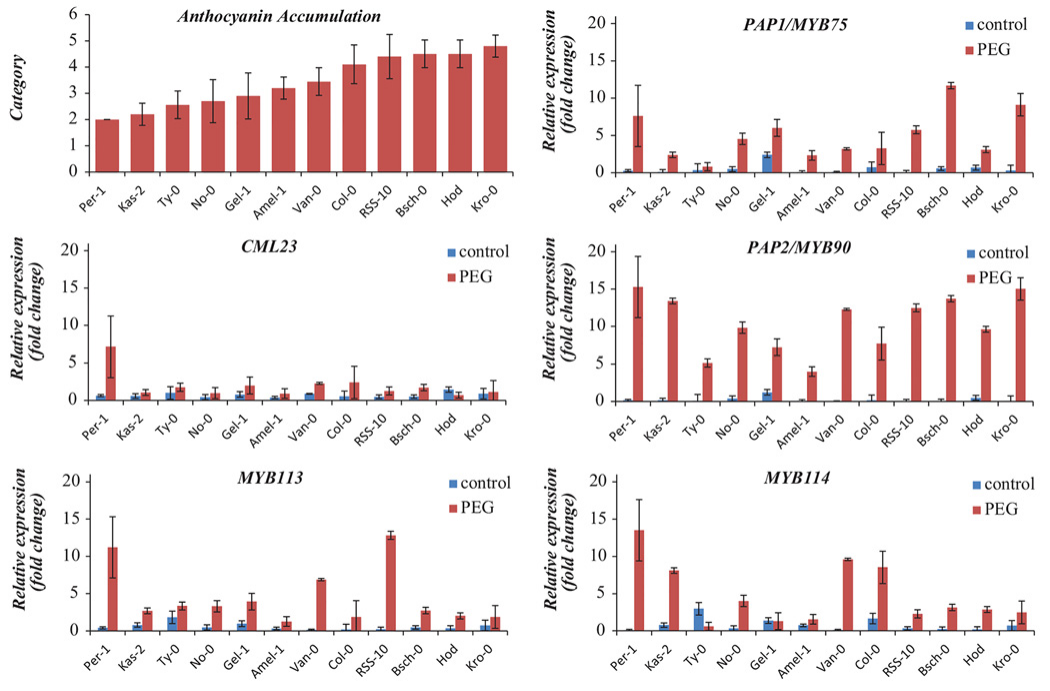
Relative expression of 4 MYB-transcription factors and CML23, AT1G44600, a calmodulin-like gene, in control and PEG-treated plants. For each gene, the values are relative to the average and are normalized using the expression of two reference genes. Error bars represent standard deviations. Accessions are ordered from low to high anthocyanin accumulation upon osmotic stress. Details can be found in S1 Data.

Natural variation can act on the level of transcription by modifying promoter regions or on the effectiveness of protein function by modifying coding regions. To assess whether any of the assigned candidate genes displayed expression variation that corresponds to differences in anthocyanin accumulation, the three MYB genes and the calmodulin-like gene at the associated locus on chromosome 1 were subjected to qPCR analysis on plants grown under control and stress conditions. The analysis included the *MYB75* transcription factor (*PAP1*, At1G56650)[29], which is phylogenetically related to the three other MYB genes and was previously shown to explain variation in anthocyanin accumulation in a recombinant inbred line (RIL) population [24]. The four MYB genes (*MYB90*, *MYB113*, *MYB114* and *MYB75*) are part of the largest class of MYB-transcription factors, the R2R3-class, and have high similarity in the overall sequence, particularly the DNA binding domains [29]. For 10 of the 12 accessions the calmoduline-like gene was not significantly up-regulated upon osmotic stress (Fig 5), suggesting that it is very unlikely that this gene is causal for the observed variation in anthocyanin accumulation. In contrast, all MYB genes were up-regulated upon osmotic stress in all accessions tested, except *MYB114*, which was not up-regulated in Ty-0. This up-regulation confirms the role of these genes in stress responses. However, for none of the genes a correlation was found between the observed anthocyanin accumulation and gene expression levels, although natural variation for anthocyanin accumulation and gene expression was detected both under control and stress conditions (Fig 5). Strikingly, *MYB90*, the candidate gene in closest proximity of the associated SNPs, showed the strongest response to the stress treatment in terms of expression, but this did not correlate with the amount of anthocyanins accumulated. These observations suggest that the causal mechanism for variation in anthocyanin accumulation is not transcriptional modification but more likely resides in functional changes. When the same 12 accessions were exposed to nitrogen deprivation, a similar trend was observed for anthocyanin accumulation (S2 Data), confirming that genetic differences are responsible for variation observed in anthocyanin accumulation and that the type of stress applied plays a minor role.

### Analysis of re-sequence data reveals a QTN in MYB90

**Fig 6:**
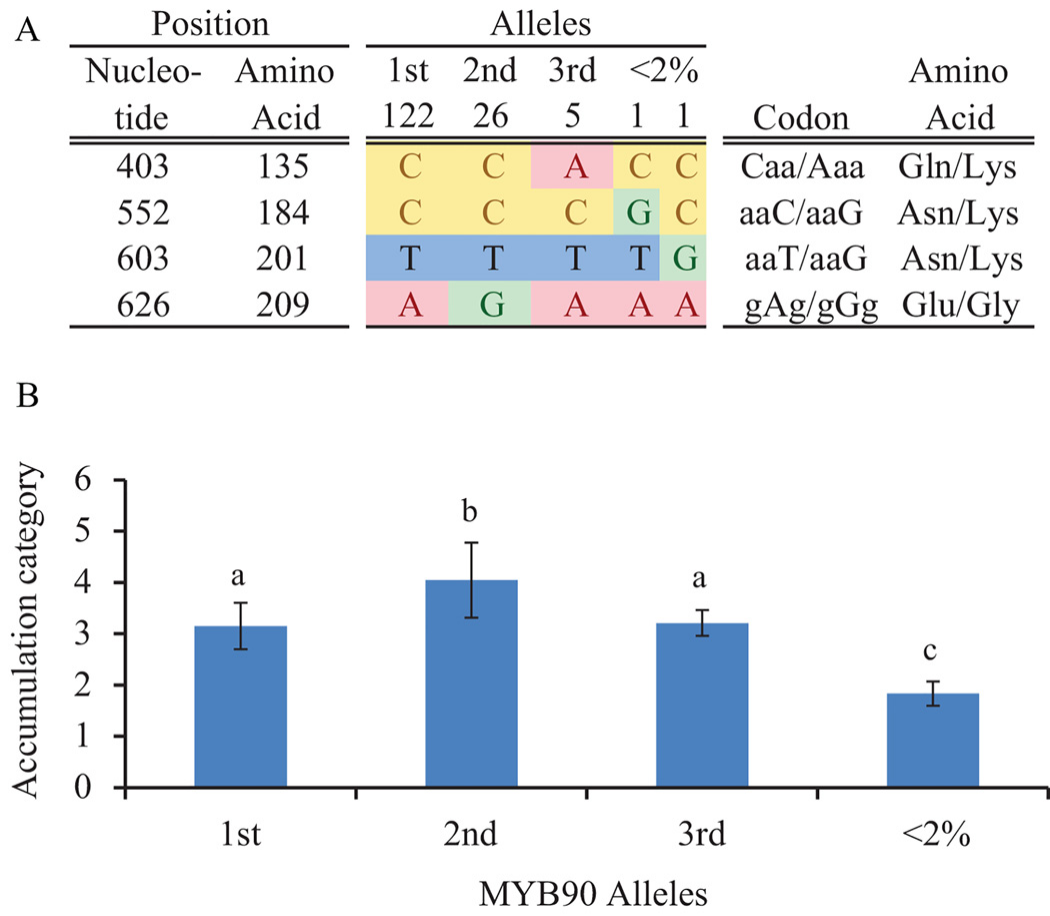
Genotypic and phenotypic differences between alleles of *MYB90*. **A)** Positions of non-synonymous SNPs and corresponding amino acid changes defining the alleles. Number of accessions carrying the allele are indicated above the double line. **B)** Anthocyanin accumulation per allele (mean +/- SD). Means per allele are compared by ANOVA following pairwise comparison using Bonferroni corrected significance threshold of α = 0.05. Letters above the bars indicate significant differences.

To gain more insight into the causal sequence variation of the MYB transcription factors, the nucleic acid and translated amino acid sequences of the phenotyped accessions for which re-sequence information was available (157 out of 360) were aligned. Because no correlation was found between the up-regulation of gene expression upon osmotic stress and the observed phenotype, the focus here was on the protein sequences and only information of the exons was taken into account (S2 Fig and Table 1). As expected, the coding sequences of the MYB family members are highly conserved, especially in the R2-R3 DNA binding domain. For each transcription factor, sequence diversity was observed with other MYB family members and also within the same gene between accessions. Because the lowest coding sequence variation was observed for *MYB90*, this gene appears to be the most conserved among the different accessions. Within the coding sequences of *MYB90* only ten mutations occurred of which 4 were non-synonymous (Table 1), whereas between 27 and 41 mutations, of which between 22 and 26 were non-synonymous, were observed for the other three MYB genes. Based on the observed non-synonymous mutations, different alleles were defined for each of the proteins. In line with the observed mutation frequencies, allelic diversity was lowest for *MYB90*, with five different alleles, whereas for the other three MYB genes, *MYB75*, *MYB113* and *MYB114*, three to four times more alleles were observed, 15, 19 and 18 alleles respectively (Table 1). For all four genes, the frequency distribution of alleles was skewed, with only a few alleles of each transcription factor dominating the population (S3 Fig). Skewed allele distributions were expected because most non-synonymous mutations will have negative effects and therefore will not be maintained in the population. In many cases the most favorable allele will dominate a population but in case of balancing selection or substitution by a newly arisen allele, i.e. a selective sweep, more than one allele can be present in a substantial part of the population. For *MYB75* clearly one allele is dominating with the most frequent allele being present in 77% of the population and the second most frequent allele in only 5% of the population. All of the other 12 alleles have allele frequencies below 5%. For the other three MYBs at least two alleles with allele frequencies above 10% occur, indicating balancing selection or allele substitution. The difference between the two most frequent alleles of the three MYBs is only one amino acid and no signs of genetic hitch-hiking were observed, indicating that the discriminating SNPs have been maintained in the population for a long time. Therefore, balancing selection is expected for *MYB90*, *MYB113* and *MYB114*. However, balancing or positive selection was not supported by Tajima’s D or Fu and Li’s D* or F* but weak purifying selection was suggested by both tests (Table 1). To test for significant differences in anthocyanin accumulation between allelic haplotypes, analysis of variance (ANOVA) was performed. Alleles with frequencies below 2% were grouped and tested against alleles with higher frequencies. Significant differences were found between alleles of *MYB*90, *MYB113* and *MYB114*, but not of *MYB75*. Subsequent pair-wise comparisons revealed significantly higher anthocyanin accumulation for the second most frequent allelic haplotype of *MYB90* compared to all other alleles (Fig 6B, allele frequency = 17%). In addition, also higher anthocyanin accumulation was observed for the second allele of *MYB114* (S4B Fig, allele frequency = 17%). This difference is significant in pairwise comparisons with most other alleles, including the first, third and fourth most frequent alleles representing 64% of the variation for *MYB114*. However, the significance threshold was not reached for three alleles which together account for 11% of the variation for *MYB114*. Not surprisingly, allele MYB90-2 and MYB114-2 are discriminated by the SNPs that were in strong linkage disequilibrium (LD) with the association peak (MYB90-2, LD = 0.70; MYB114-2, LD = 0.65). Allele MYB90-2 is the only allele that contains a glycine instead of a glutamic acid at AA position 210 (Fig 6A), whereas allele MYB114-2 is the only allele carrying a stop-codon at AA position 141 and the protein is, therefore, truncated (S4A Fig). All other *MYB114* alleles do not contain this pre-mature stop-codon and their encoded proteins have the same size as the other MYB-protein family members. Overexpression of allele MYB114-2, resulting in truncated proteins, did not result in increased anthocyanin accumulation [16]. The SNP discriminating this allele from the other alleles is, therefore, most probably not causal for the detected association. The two discriminating polymorphisms in *MYB90* and *MYB114* are highly linked (LD = 0.93). All accessions, except two, that contain allele MYB114-2 also contain allele MYB90-2. For *MYB113*, significant differences were observed between the first two alleles, but both are not significantly different from all other alleles. The SNP that is discriminative between those two alleles is also polymorphic in other alleles of *MYB113* and can, therefore, not explain the strong association detected by GWA mapping. Interestingly, significant lower anthocyanin accumulation was observed for the two alleles of *MYB90* with low (<2%) allele frequency. These two alleles were present in two accessions, Kas-2 and Per-1, of which the very low anthocyanin accumulation upon osmotic stress was confirmed in the second experiment used to determine gene expression. This suggests that some rare mutations in *MYB90* or its cis-regulatory regions affect anthocyanin accumulation more severe than the mutation at position 210. Possibly, such severe reduction in induced anthocyanin accumulation is not favorable in any environment and therefore these alleles do not occur at higher allele frequencies and are purged from the population. In conclusion, because differences in gene expression could not explain the observed phenotype and given the allelic differences observed, the polymorphism in *MYB90*, discriminating the second allele from the rest, seems to be causal for the associations detected in GWA mapping. However, the SNP discriminating the first two alleles of *MYB114* was found to be linked to both the polymorphism in *MYB90* and the associated SNP of the GWA mapping. This tight linkage cannot rule out a joint role in the natural variation in anthocyanin accumulation for allele MYB114-2 and allele MYB90-2 completely.

**Table 1:** Statistics of the sequence diversity of *MYB75*, *MYB113* and *MYB114*, *MYB90* and results of tests for deviations from neutrality of selection of Tajima [30] and Fu and Li [31].

		SNPs			Fu and Li		
gene	mutations	syn	non-syn	Tajima's D	D*	F*	# of alleles
MYB75	36	13	21	-1.32	-2.00^&^	-2.02^&^	15
MYB113	41	13	25	-2.05*	-3.16*	-3.00*	19
MYB114	27	4	22	-1.18	-2.17^&^	-2.23^&^	18
MYB90	10	6	4	-1.14	-6.94*	-5.96*	5

SNP = single nucleotide polymorphism; mutation: this number deviates from the number of SNPs if at one position more than two different nucleotides are observed; syn = synonymous, non-syn = non-synonymous. Significance is indicated by * (α = 0.05) and ^&^ (α = 0.10).

## Discussion

### Natural variation in MYB90 is causal for anthocyanin accumulation

Variation for constitutive and osmotic stress induced anthocyanin accumulation was observed within natural accessions of Arabidopsis. Osmotic stress induced accumulation resulted in extreme variation, ranging from completely green to completely red/purple plants. Using a GWA mapping approach, a region on chromosome 1 explaining more than 40% of the observed variation was identified. Analysis of the alleles of three MYB-transcription factors in this region, known to be involved in anthocyanin biosynthesis, allowed us to identify two SNPs, one in *MYB90* at AA position 210 and one in *MYB114* at AA position 141, which were discriminative for gene alleles that were associated with higher anthocyanin accumulation. The biosynthesis pathway of anthocyanins reveals that these MYB transcription factors upregulate the genes in the last part of the anthocyanin biosynthesis pathway (S1 Fig, [15]). Simultaneous knock-down by RNAi of all four MYB transcription factors involved in the later steps of the pathway results in anthocyanin deficiency under stress [16]. Overexpression studies of the four MYBs revealed that the up-regulation of either one of them is sufficient to increase anthocyanin accumulation in young leaves under control conditions and upon osmotic stress treatment [16, 18]. *MYB114* is truncated in Col-0, due to a mutation at AA position 141, which introduces a pre-mature stopcodon. Overexpression of the truncated protein, corresponding to allele MYB114-2, does not lead to increased anthocyanin accumulation, whereas overexpression of the full protein, corresponding to allele MYB114-1, results in higher levels of anthocyanins [16]. This observation is in contrast with our results that indicate that the truncated allele MYB114-2 is associated with higher anthocyanin accumulation and allele MYB114-1 with lower accumulation. We, therefore, postulate that the allelic differences in *MYB114* are not causal for the observed variation in anthocyanin accumulation in wild accessions. This leaves the allelic variation in *MYB90* as the cause to explain stress-induced anthocyanin accumulation in natural accessions. Because the causal polymorphism is located at the end of the protein outside the DNA-binding domain we speculate on an enhanced functional activity of the modified protein. However, because of the tight linkage between *MYB114* and *MYB90* we cannot rule out a genetic interaction between the two transcription factors.

### GWA mapping in natural populations reveals evolutionary significance of variation

GWA mapping and subsequent analysis of alleles of candidate genes using re-sequence information of a large number of accessions resulted in the identification of a single gene (*MYB90*) to be responsible for almost half of the variation present in anthocyanin accumulation in natural populations. Classical QTL mapping studies in three different biparental experimental populations also reported natural variation in anthocyanin accumulation induced by stress [24–26]. In one of these studies, anthocyanin accumulation in seedlings of a RIL population derived from a cross between the accessions Landsberg *erecta* (L*er*) and Cape Verde Islands (Cvi) was induced by high sucrose levels [24]. In another study, RILs derived from a cross between the accessions Bayreuth-0 (Bay-0) and Shahdara (Sha) were grown under low nitrogen conditions and as a result displayed increased levels of anthocyanins [25]. Finally, variation for anthocyanin accumulation was observed upon cold acclimation in the L*er* x Eri-1 population [26]. These three QTL studies detected a major QTL on chromosome 1 and sequence variation in MYB75 was proven to result in differences in anthocyanin accumulation [24, 26]. It is suggested that besides MYB75 also MYB90 could contribute to this major QTL [26]. GWA mapping of our data did not detect the slightest association of *MYB75* with anthocyanin accumulation. The different results of these two approaches (i.e. experimental vs natural population mapping) poses questions about the biological relevance of the identified variants and the impact on selection processes in natural populations. In our study similar patterns of anthocyanin accumulation were observed upon induction by osmotic stress and nitrogen deprivation, suggesting that the stress response is governed by genetic variation rather than by the type of stress applied.

Available re-sequence data identified the causal GWA SNP at AA position 210 of *MYB90*. This SNP substituted a glycine for glutamic acid in 17% of the accessions analysed. Because this is the only SNP discriminating allele MYB90-2 from all other alleles, it has probably been present in the population for a long time, suggesting balancing selection between the haplotypes with the glycine and haplotypes with the glumatic acid residue. However, the accessions L*er*, Cvi, Bay-0 and Sha all belong to the glutamic acid haplotype and hence this locus is not segregating in the RIL populations derived from these lines and no QTL could be detected due to the lack of variation in *MYB90*. Re-sequence data of Eri-1 are not available, but it was suggested that this accessions has a different haplotype than L*er* [26].

In contrast, allele specific analysis of *MYB75* revealed that 77% of the natural accessions share the most common allele while the other 23% are represented by 14 different alleles with allele frequencies of 5% or less. This suggests that variation in *MYB75* is the result of random independent mutation events without a clear selection for, or against, a specific allele. Unfortunately, re-sequence data for *MYB75* of Sha and Bay-0 are incomplete with missing data at many positions and Eri-1 data are not available yet. However, L*er* and Cvi each carry a unique allele and accumulate anthocyanins at high and low levels, respectively. Segregation at this locus, therefore, results in the detection of a strong QTL at this position in the *Ler* x Cvi RIL population. GWA mapping failed to detect *MYB75* as a causal locus explaining variation in anthocyanin accumulation because of the high number of rare alleles, of which many may be functionally indifferent, at this locus. The power to detect a rare allele using GWA is rather low especially if the population size is limited. In addition, rare alleles will be highly linked to other rare alleles resulting in high false discovery rates [32]. As a consequence SNPs with low allele frequency are excluded in most GWA mapping studies. RIL populations are ideal to find such rare causal alleles, but only a very limited amount of the variation that can be found in nature can be tested [33]. In contrast, GWA mapping assesses most variation present in nature and will detect causal genes that are relevant in natural settings because different alleles are present in a substantial part of the population.

### MYB75 and MYB90 cannot account for all observed variation

The almost normal distribution of the values of stress-induced anthocyanin accumulation in the natural population indicates that this trait is complex, meaning that the expression and regulation of several genes together result in the observed phenotype. In our GWA study only one region was found to associate with induced anthocyanin accumulation. The causal polymorphism could explain 40% of the phenotypic variation, corresponding to one accumulation category difference (S2 Table). However, differences larger than one accumulation category were observed and confirmed between natural accessions. The other 60% of the phenotypic variation may be caused by random variation, but also by genes with small effects or by alleles with allele frequencies lower than 5% that were excluded from the analysis. Clear examples are accessions Kas-2 and Per-1, that have a unique allele of *MYB90* and both show strong deficiency in anthocyanin accumulation upon osmotic stress. That anthocyanin accumulation is not a monogenic trait is also confirmed in QTL mapping studies. Besides the major QTL on chromosome one, also other small [24, 34] or large effect [26] QTLs were detected in the bi-parental studies. This suggests that besides the two key-players, *MYB90* and *MYB75*, most probably more regulatory genes are involved, which have a small effect size compared to *MYB90* and *MYB75*.

### Allele frequencies of MYB90 are explained by local adaptation

Anthocyanin accumulation can be induced by many different stresses and in many cases anthocyanins have been shown to help plants cope with stress [3–5, 35], (S2 Data). It is reported that anthocyanins protect against high light and ROS [8, 9], that they can bind to metals [10] and that they function in regulating the osmotic potential within plant tissues [11]. Because of these functions, the production of anthocyanins as a response to stress is a beneficial trait for plants in many climates. However, anthocyanin production is an energy-demanding process and therefore strong natural selection against their accumulation is expected [36]. The large natural variation observed in this study suggests that anthocyanin production is part of the local adaption process. Allele MYB90-2, which was associated with higher anthocyanin accumulation, is present in almost 17% of the population. The presence of two alleles at high frequency in the world-wide population suggests balancing selection. Allele MYB90-2 might be favored in some environments, whereas the other alleles might be favored in other environments. The large geographical distribution of the different alleles also suggests that the selection pressure for stress-induced anthocyanin accumulation is not specific to one particular environment but widespread.

Interestingly, anthocyanins not only accumulate in response to stress but are also produced in plants grown under control conditions. Even though the accumulation of anthocyanins was much lower under control conditions, variation between accessions was consistent. For almost 100 accessions, 25% of the population, all three replicates were assigned to accumulation category two, whereas for 135 accessions, 37% of the population, no anthocyanin accumulation was observed in any replicate. Constitutive production of anthocyanins can be beneficial in local climates where stress occurs on regular basis e.g. daily or annually, and therefore can be anticipated. For example, in plants grown in equatorial regions with high UV-B levels [2] or in winter-annuals that have to cope with low temperatures in autumn and winter [12]. However, no association with variation in anthocyanin accumulation under control conditions was detected, suggesting that natural selection for constitutive anthocyanin production does not act on a limited number of genes or has evolved independently in many accessions. A very weak correlation was found between the accumulation of anthocyanins under control and stressed conditions. This further suggests that the selection criteria and targets for constitutive and induced accumulation are different.

For both control and stress conditions, a very weak correlation was found between anthocyanin accumulation in different accessions and the latitude of their origin of collection. Further comparisons of the geographical distribution did not reveal additional geographical trends. This is in contrast with a study of anthocyanin accumulation during autumn leaf senescence in deciduous species, in which a clear latitudinal cline, correlated with UV-B radiation, was observed [2]. This emphasizes the fact that anthocyanins have many different functions within and between species, and therefore may be positively selected for in several different environments.

## Conclusion

This GWA study reveals that sequence variation in a small 15 kb region on chromosome 1 is the basis of a large part of the observed natural variation in anthocyanin accumulation induced by osmotic stress. Using re-sequence data and expression studies, the causal SNP was identified at amino acid position 210 of *MYB90*. The two most frequent alleles of *MYB90* differ only at this position. These two alleles dominate the population and in less than 5% of the population another allele was observed. This suggests balancing selection between the two alleles. Analysis of the geographical origin of the studied accessions suggests that the macro climate is not the driving force behind positive or negative selection for induced and constitutive anthocyanin accumulation. We assume, therefore, that local climate differences are determining the allelic constitution of natural accessions. These results emphasize that GWA mapping is very powerful in finding sequence variation that plays a major role in natural selection.

## Material and Methods

### Plant material

For association mapping a collection of 360 accessions selected to capture most of the genetic variation present within the species *Arabidopsis thaliana* was used [37–39]. Each accession is genotyped with approximately 215k SNP markers (Col vs non-Col) [22, 40]. For confirmation of results a selection of 12 accessions from this collection was grown representing the whole range of anthocyanin accumulation: cs76210, cs28786, cs76150, cs28014, cs28279, cs28564, cs22689, cs76113, cs76297, cs28099, cs28420, cs76141. The same twelve accessions were subjected to nitrogen deprivation.

### Experimental design

Association mapping: Plants were grown in six replicate blocks. Each accession was present once in each block. Within each block the plants were grown in nine trays each containing 40 plants. Within a tray the plants had a fixed position. The nine trays were positioned randomly within a block.

In the confirmation experiment the plants were grown in four trays each containing 40 plants. Each tray was treated as a block. In each block all twelve accessions were replicated at least 3 times in a randomized design.

In the nitrogen deprivation experiment the twelve accessions were grown in three larger trays. In each tray all twelve accessions were replicated 5 times in a randomized design.

### Growth conditions

Seeds were sown in petri-dishes on wet filter paper. After four days of cold treatment, they were placed in the light at room temperature for 1.5 day to germinate. Germinated seeds were placed on rockwool blocks saturated with nutrient solution (Hyponex, 1mM N, 1.1 mM P, 5.9 mM K). Three times a week the perforated trays containing the rockwool blocks were placed in nutrient solution for approximately five minutes. For the osmotic stress treatment, the trays were fed with nutrient solution containing 0.1 g/ml poly ethylene glycol (PEG8000) on day 8, 11, 13 and 15. Control blocks were fed with nutrient solution only.

For the association mapping experiment, three blocks received PEG treatment and three blocks received control treatment. In the confirmation experiment, three blocks received PEG treatment and 1 block received control treatment.

In the nitrogen deprivation experiment, plants in the first tray received Hyponex solution containing 4.1 mM NO_3_ and 1.7 mM NH_4_ (control condition), in the second tray 1/5 of the nitrogen supply (0.82 mM NO_3_ and 0.34 mM NH_4)_ and in the third tray 1/10 of the nitrogen supply (0.41 mM NO_3_ and 0.17 mM NH_4_ (1/10 nitrogen). The plants were watered with the appropriate nitrogen solution twice a week. After four weeks, pictures were taken and the rosettes were scored for anthocyanin accumulation (S2 Data).

### Quantification of anthocyanin accumulation and RNA extraction

On day 27 (GWA experiment) or day 21 (confirmation experiment), each plant was scored visually for anthocyanin accumulation. Five categories were considered, ranging from 1 (totally green plant) to 5 (totally red/purple plant) (Fig 1A). Frozen rosette tissue was grinded with a mortar and pestle. RNA was extracted from 10–15 mg tissue [41]. Anthocyanins were extracted according to the procedure described by [42]. In brief, 50–200 mg tissue was incubated overnight in 300 μl of methanol containing 1% fuming HCl at 4°C in the dark. Thereafter 200 μl MilliQ water and 500 μl chloroform were added. After centrifugation (14,000*g*, 5 min) 400 μl of the aqueous top layer was taken and 400 μl 60% methanol containing 1% HCl was added. Anthocyanin content was determined spectrophotometrically as the difference between absorbance at 530 nm and 657 nm.

### cDNA synthesis, qPCR and data analysis

For plants of the confirmation experiment, gene expression of *MYB75* (*PAP1*), *MYB90* (*PAP2*), *MYB113*, *MYB114*, *CML23 (AT1G66400)*, *(ANS (AT4G22880)*, *DFR(AT5G42800)*, *and UFGT (AT4G14090)*was determined by qPCR in control and PEG-treated plants. Three biological and 2 technical replicates were analysed. RNA concentration was determined using Nanodrop ND-1000 (Nanodrop Technologies Inc.) cDNA was synthesized from 500 ng RNA using iScript™ cDNA Synthesis Kit (Biorad). cDNA samples were diluted 10× with sterile milliQ water. For each qPCR, 2.5 μl of cDNA, 5 μl of iQ SYBR Green Supermix (Bio-Rad) and 0.25 μl of primer mix (from a 10 μM working solution of forward and reverse primer) was added and supplemented with water to a final volume 10 μl. The RT–qPCRs were run on a CFX96 (Bio-Rad). The qPCR program consisted of a first step at 95°C for 3 min and afterwards 40 cycles alternating between 15 s at 95°C and 30 s at 60°C. Routinely, a melting curve analysis was performed after the qPCR run (between 55 and 95°C with 0.5°C increments for 10 s each). AT2G28390, AT1G18610, AT4G26410, AT4G34270 and AT1G13320 were found to be appropriate reference genes and the last three are used for normalization (Details S1 Table and S1 Data) [43, 44]. See S1 Table for primer sequences. Primer efficiency was determined from the linear part of the amplification curves using the program LinregPCR [45]. Relative expression values were determined using the program qBase+ (Biogazelle).

### Geographical distribution

The latitude and longitude of the place of origin of all accessions was obtained from the ABRC website.

### Statistical analysis

GWAS was performed using the EmmaX software package, based on [46, 47]. A mixed model was used which corrects for population structure based on the identity-by-state matrix (also called kinship matrix) of all SNPs. SNPs with an allele frequency lower than 0.05 were excluded from the analysis. Significance was determined using the Bonferroni correction for multiple testing. Conditional GWAS was performed using the on-line GWApp tool [28]. The most significant SNP (chr1, position 24769177) was used as co-factor. A significance threshold of α = 0.05 was used after Bonferroni-correction for multiple testing. Broad-sense heritabilities were calculated as H^2^ = V_g_/(V_g_+V_e_) where V_g_ is the between accessions variation and V_e_ is the within accessions variation. Spearman’s rank correlation coefficient (ρ) (2-tailed, α = 0.05) (SPSS, [48]) was used to determine correlation between data series.

### Sequence analysis and definition of alleles

The sequences of *MYB75*, *MYB113*, *MYB114* and *MYB90* were extracted from the 1001 genome browser for all accessions for which data were available (157 accessions, for list see S1 Table). For all further analyses only the exon-sequences were used. If an accession contained missing data in an exon it was removed from the analysis of the corresponding protein. For each MYB transcription factor, alleles were defined based on non-synonymous SNP diversity. The most common allele was classified 1^st^, the second most frequent 2^nd^ etc. Deviations from neutrality were tested by calculating Tajima’s D [30] and Fu and Li’s D* and F* [31] using DNASP software, version 5 [49].

## Supporting Information

S1 Data Expression data of 8 genes in 12 accessions grown in control and osmotic stress conditions. Genes: ANS (AT4G22880), DFR(AT5G42800), UFGT (AT4G14090), CML23 (AT1G66400), PAP1(AT1G56650), PAP2 (AT1G66390), MYB113 (AT1G66370), MYB114 (AT1G66380). Accessions: Per-1, Kas-2, Ty-0, No-0, Gel-1, Amel-1, Van-0, Col-0, RSS-10, Bsch-0, Hod, Kro-0.
(XLSX)

S2 Data Anthocyanin accumulation in 12 accessions upon moderate (20% of control) and severe (10% of control) nitrogen deprivation. Accessions: Per-1, Kas-2, Ty-0, No-0, Gel-1, Amel-1, Van-0, Col-0, RSS-10, Bsch-0, Hod, Kro-0.
(XLSX)

S1 Fig Biosynthesis pathway of anthocyanins and flavonols (simplified from [15]). The early biosynthesis genes are regulated by MYB transcription factors. The late biosynthesis genes are regulated by a complex of two MYB-, two bHLH- and one W40-transcription factor. Enzyme and gene abbreviations are as follows: PAL, phenylalanine ammonia lyase; C4H, cinnamic acid 4-hydroxylase; 4CL, 4 coumarate CoA ligase; CHS, chalcone synthase; CHI, chalcone isomerase; F3H, flavanone 3-hydroxylase; DFR, dihydroflavonol reductase; FLS, flavonol synthase; ANS/LDOX, anthocyanidin synthase/leucoanthocyanidin dioxygenase; UFGT, UDP-flavonoid glucosyl transferase; *MYB11*, At3g62610; *MYB12*, At2g47460; *MYB111*, At5g49330; *TTG1*, At5g24520; *TTL8*, At4g09820; *GL3*, At5g41315; *EGL*, At1g63650; *PAP1*, *MYB75*, At1g56650; *PAP2*, *MYB90*, At1g66390; *MYB113*, At1g66370; *MYB114*, At1g66380.
(PDF)

S2 Fig Comparison of sequence diversity between and within four MYB family members, *MYB75*, *MYB113*, *MYB114*, *MYB90*. **A** Alignment of amino acid sequences of *MYB75* (*PAP1*), *MYB90* (*PAP2*), *MYB113*, and *MYB114*. Green and blue high-lights indicate respectively, synonymous and non-synonymous differences in the AA codon of the MYB indicated in the first column between accessions. The most frequent AA codon is represented (NB the most frequent AA codon will in some cases not represent Col-0). Red letter indicates that the amino acid at that position deviates from the common (conserved) amino acid present in the other MYB-proteins. Box around the letter indicates a SNP in the codon that is in strong LD (r^2^>0.65) with the highest associated SNP found in the GWAS.
(PDF)

S3 Fig Frequency distribution of alleles of four MYB transcription factors, *MYB75*, *MYB113*, *MYB114* and *MYB90*, in the population. The alleles are ordered from most to least frequent and alleles with a frequency lower than 2% are pooled together.
(PDF)

S4 Fig Alleles of *MYB114* A Positions of non-synonymous SNPs and corresponding amino acid changes defining the alleles. Number of accessions carrying the allele are indicated above the double line. **B** Anthocyanin accumulation per allele (mean +/- SD). Means per allele are compared by ANOVA following pairwise comparison using Bonferroni corrected significance threshold of α = 0.05. Letters above the bars indicate significant differences.
(PDF)

S1 Table Primers used for qPCR. (PDF)

S2 Table SNPs significantly associated with the accumulation of anthocyanin in stress conditions. (PDF)
