# Supplementary figures and images for: GWA Mapping of Anthocyanin Accumulation Reveals Balancing Selection of MYB90 in Arabidopsis thaliana
Source: PLoS One. 2015 Nov 20;10(11):e0143212. doi: 10.1371/journal.pone.0143212 (PMC4654576; doi:10.1371/journal.pone.0143212)

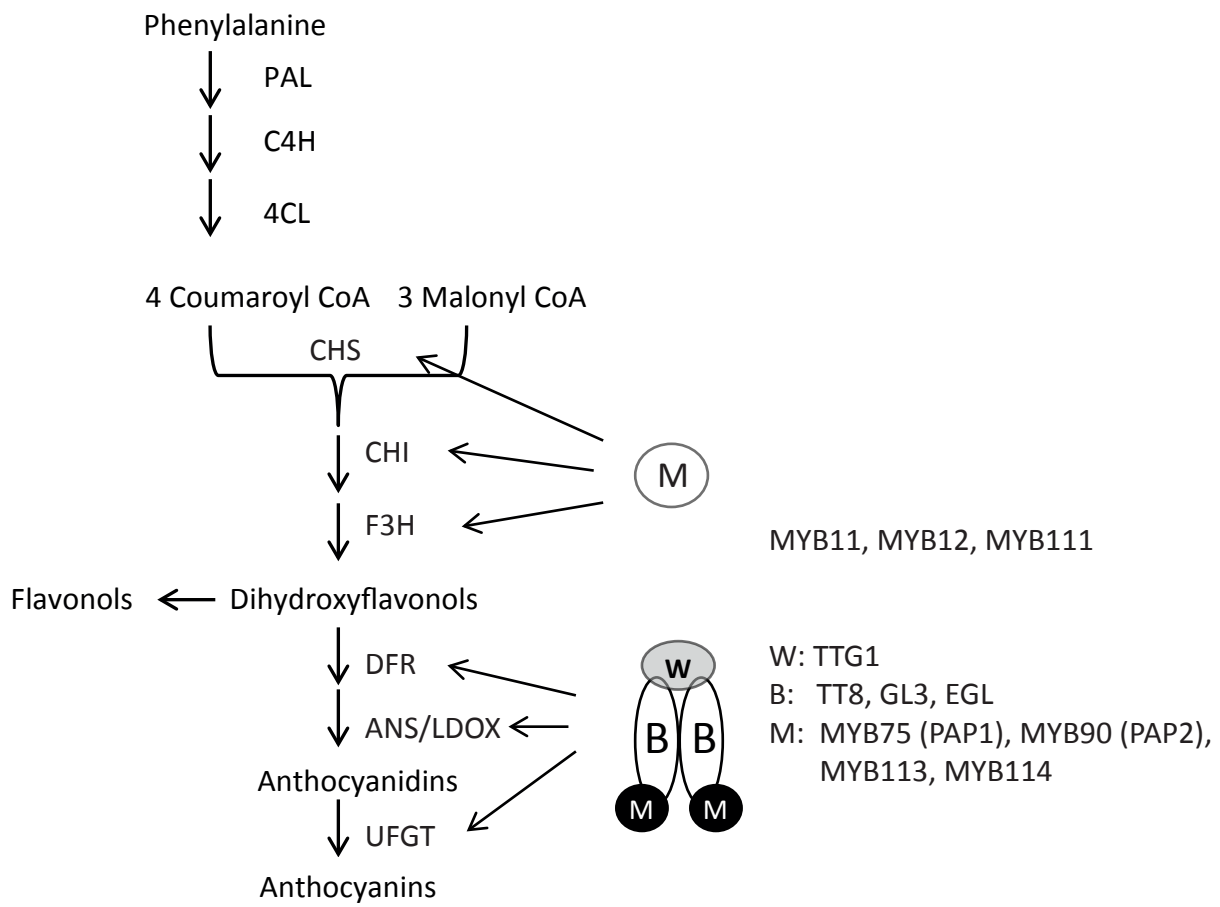

Supplement: S1 Fig — The early biosynthesis genes are regulated by MYB transcription factors. The late biosynthesis genes are regulated by a complex of two MYB-, two bHLH- and one W40-transcription factor. Enzyme and gene abbreviations are as follows: PAL, phenylalanine ammonia lyase; C4H, cinnamic acid 4-hydroxylase; 4CL, 4 coumarate CoA ligase; CHS, chalcone synthase; CHI, chalcone isomerase; F3H, flavanone 3-hydroxylase; DFR, dihydroflavonol reductase; FLS, flavonol synthase; ANS/LDOX, anthocyanidin synthase/leucoanthocyanidin dioxygenase; UFGT, UDP-flavonoid glucosyl transferase; MYB11, At3g62610; MYB12, At2g47460; MYB111, At5g49330; TTG1, At5g24520; TTL8, At4g09820; GL3, At5g41315; EGL, At1g63650; PAP1, MYB75, At1g56650; PAP2, MYB90, At1g66390; MYB113, At1g66370; MYB114, At1g66380. (PDF) [file pone.0143212.s003.pdf]

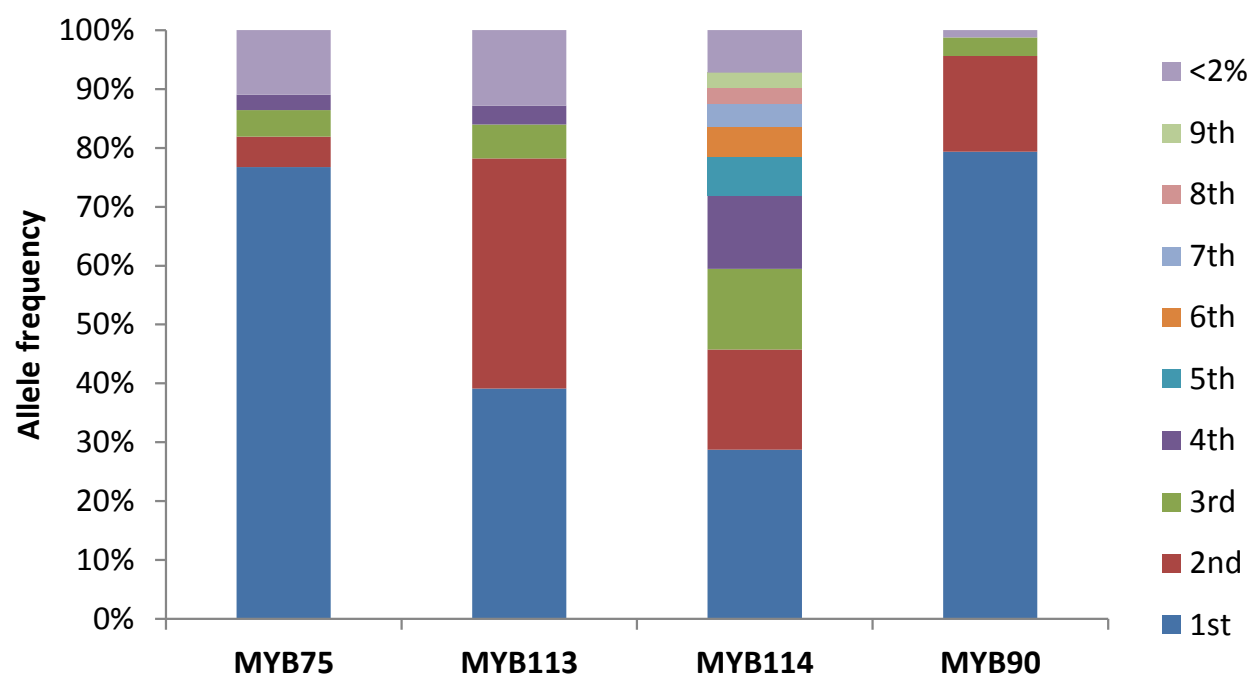

Supplement: S3 Fig — The alleles are ordered from most to least frequent and alleles with a frequency lower than 2% are pooled together. (PDF) [file pone.0143212.s005.pdf]
