# Supplementary material for: GWA Mapping of Anthocyanin Accumulation Reveals Balancing Selection of MYB90 in Arabidopsis thaliana
Source: PLoS One. 2015 Nov 20;10(11):e0143212. doi: 10.1371/journal.pone.0143212 (PMC4654576; doi:10.1371/journal.pone.0143212)
Supplement: S2 Fig — A Alignment of amino acid sequences of MYB75 (PAP1), MYB90 (PAP2), MYB113, and MYB114. Green and blue high-lights indicate respectively, synonymous and non-synonymous differences in the AA codon of the MYB indicated in the first column between accessions. The most frequent AA codon is represented (NB the most frequent AA codon will in some cases not represent Col-0). Red letter indicates that the amino acid at that position deviates from the common (conserved) amino acid present in the other MYB-proteins. Box around the letter indicates a SNP in the codon that is in strong LD (r2>0.65) with the highest associated SNP found in the GWAS. (PDF) [file pone.0143212.s004.pdf]

```
MYB75      1   MEGSSKGLRK GAWTTEEDSL LRQCINKYGE GKWHQVPVRA GLNRCRKSCR LRWLNYLKPS
MYB113     1   MGESPKGLRK GTWTTEEDIL LRQCIDKYGE GKWHRVPLRT GLNRCRKSCR LRWLNYLKPS
MYB114     1   MEGSSKGLRK GAWTAEEDSL LRQCIGKYGE GKWHQVPLRA GLNRCRKSCR LRWLNYLKPS
MYB90      1   MEGSSKGLRK GAWTAEEDSL LRLCIDKYGE GKWHQVPLRA GLNRCRKSCR LRWLNYLKPS
                                               R2

MYB75      61  IKRGKLSSDE VDLLLRLHRL LGNRWSLIAG RLPGRTANDV KNYWNTHLSK KHE~PCCKIK
MYB113     61  IKRGKLCSDE VDLVLRLHKL LGNRWSLIAG RLPGRTANDV KNYWNTHLSK KHDERCCKTK
MYB114     61  IKRGKFSSDE VDLLLRLHKL LGNRWSLIAG RLPGRTANDV KNYWNTHLSK KHE~PCCKTK
MYB90      61  IKRGRLSNDE VDLLLRLHKL LGNRWSLIAG RLPGRTANDV KNYWNTHLSK KHESSCCKSK
                                          R3

MYB75      121 MKKRDITPIP TTPALKNNVY KPRPRSFTVN NDCNHLNAPP KVDVNPPCLG LNINNVCDNS
MYB113     121 MINKNITSHP TSSAQKIDVL KPRPRSFSDK NSCNDVNILP KVDVVPLHLG LNNNYVCESS
MYB114     121 IKRINIITPP NTPAQKVDIF KPRPRFFSIK TGCNHLDGQS EVGVIPPCLG LDNDNVCENS
MYB90      121 MKKKNIISPP TTPVQKIGVF KPRPRSFSVN NGCSHLNGLP EVDLIPSCLG LKKNNVCENS

MYB75      181 IIYNKDKKKD QLVN~NLIDG DNMWLEKFLE ESQEVDILVP EATTTEKGDT LAFDVDQLWS
MYB113     181 ITCNKDEQKD KLININLLDG DNMWWESLLE ~~~~ADVLGP EATETAKGVT LPLDFEQIWA
MYB114     181 ITCNKDDEKD DFVD~NFMVG DNIWLERLLD EGQEVDVLVT EAAATEKEGT LAFDVEQLWN
MYB90      181 ITCNKDDEKD DFVN~NLMNG DNMWLENLLE ENQEADAIVP EATTAEHGAT LAFDVEQLWS

MYB75      241 LFDGETVKFDX
MYB113     241 RFDEETLELNX
MYB114     241 LFDGETVIFDX
MYB90      241 LFDGETVELDX
```
