# Supplementary material for: GWA Mapping of Anthocyanin Accumulation Reveals Balancing Selection of MYB90 in Arabidopsis thaliana
Source: PLoS One. 2015 Nov 20;10(11):e0143212. doi: 10.1371/journal.pone.0143212 (PMC4654576; doi:10.1371/journal.pone.0143212)

Position		Alleles											
Nucleo-tide	Amino Acid	1st 44	2nd 26	3rd 21	4th 19	5th 10	6th 8	7th 6	8th 4	9th 4	<2% 11	Codon	Amino Acid
41	12	C	C	G	C	C	C	C	C	C	C	aCt/aGt	Thr/Ser
65	20	G	G	G	G	G	G	G	G	G	G A	aGg/aAG	Arg/Lys
103	33	C	C	C	C	C	G	C	C	C	C	Caa/Gaa	Gln/Glu
237	78	G	G	G	G	G	G	G	G	G	G T	aaG/aaT	Lys/Asn
391	129	A	A	A	A	A	A	A	A	A	A G	ACa/GCa/ATa	Thr/Ala/Ile
392		C	C	C	C	C	C	C	C	C	C T		
395	130	C	C	C	T	C	C	T	C	T	C T	cCg/cTg	Pro/Leu
405	134	A	A	A	A	A	A	A	C	A	A	aaA/aaC	Lys/Asn
418	138	A	T	A	A	A	A	A	A	A	A	Tag/Aag	Stop/Lys
442	146	A	A	A	A	C	A	A	A	A	A	Aat/Cat	Asn/His
463	153	C	C	C	C	C	C	T	C	C	C T	Ctc/Ttc	Leu/Phe
481	159	G	G	G	G	G	G	G	G	G	G A	Gtt/Att	Val/Ile
487	161	G	G	G	G	G	G	G	G	G	T G	Gta/Tta	Val/Leu
497	164	C	C	C	C	C	C	C	C	C	T C	cTa/cCa	Leu/Pro
523	173	G	G	G	G	G	G	G	G	G	G A	Gtt/Att	Val/Ile
548	181	A	A	A	A	A	A	A	A	A	A G	aAc/aGc	Asn/Ser
613	203	C	C	C	G	C	C	G	C	C	C G	CCt/GGt/CGt	Pro/Gly/Arg
614		G	G	G	G	G	G	G	G	G	G C		
629	208	G	G	G	G	G	G	G	G	G	G A	gAc/gGc	Asp/Gly
664	220	G	G	G	G	G	G	G	G	G	G A	Gca/Aca	Ala/Thr
670	222	G	G	G	G	A	G	G	G	G	G	Gaa/Aaa	Glu/Lys
678	225	G	G	G	G	G	G	G	G	G	G C	gaG/gaC	Glu/Asp

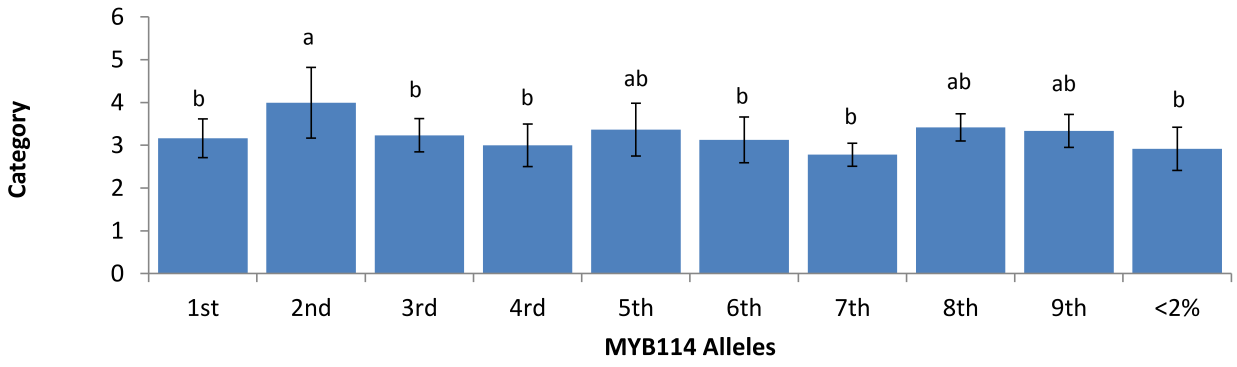

Supplement: S4 Fig — Number of accessions carrying the allele are indicated above the double line. B Anthocyanin accumulation per allele (mean +/- SD). Means per allele are compared by ANOVA following pairwise comparison using Bonferroni corrected significance threshold of α = 0.05. Letters above the bars indicate significant differences. (PDF) [file pone.0143212.s006.pdf]
