# Supplementary material for: GWA Mapping of Anthocyanin Accumulation Reveals Balancing Selection of MYB90 in Arabidopsis thaliana
Source: PLoS One. 2015 Nov 20;10(11):e0143212. doi: 10.1371/journal.pone.0143212 (PMC4654576; doi:10.1371/journal.pone.0143212)
Supplement: S1 Table — (PDF) [file pone.0143212.s007.pdf]

Table S1 Primers used for qPCR

Gene		Sequence (5' to 3')	Remark
AT2G28390	F	AACTCTATGCAGCATTTGATCCACT	Reference gene[ab]
	R	TGATTGCATATCTTTATCGCCATC	
AT1G18610	F	CTCAGGTTGATGAAGCGTCT	Reference gene[a]
	R	AAAACCCACCCTATCTCCAG	
AT4G26410	F	GAGCTGAAGTGGCTTCCATGAC	Reference gene[a]
	R	GGTCCGACATACCCATGATCC	
AT4G34270	F	GTGAAAACTGTTGGAGAGAAGCAA	Reference gene[a]
	R	TCAACTGGATACCCTTTCGCA	
AT1G13320	F	TAACGTGGCCAAAATGATGC	Reference gene[ab]
	R	GTTCTCCACAACCGCTTGGT	
AT1G56650	F	TCTTCTTCTTCGCCTTCATAG	*MYB75, PAP1*
	R	ATCTTACAACACGGTTCATG	
AT1G66370	F	TCCGATGAAGTTGATCTTG	*MYB113*
	R	TCATCGTGCTTCTTACTCAA	
AT1G66380	F	GTCAAGAACTACTGGAACACC	*MYB114*[c]
	R	ATATCGACTTTTTGGGCC	
AT1G66390	F	CAAGAGAGGAAGACTTAGCAA	*MYB90, PAP2*
	R	GATGGGTGTTCCAGTAATTT	
AT5G42800	F	AGGCCAAAATACCCCGAA	*DFR*
	R	TAAACCCCATGTCCGTCAG	
AT4G22880	F	GTGGACAATTGGAATGGGAAGA	*ANS*
	R	CCGACAGAGAGAGCCTTGA	
AT4G14090	F	TTCCGGTGGTTGCGTTTC	*UFGT*[c]
	R	TCCACATCTCCTTCCTCCCC	
AT1G66400	F	CAAAATCAACACAACCATCACC	*CML*[c]
	R	AAGGATCTCGGCTAATGAGT	

[a]*Reference gene selected from Czechowski et al. 2005*

[b]*Reference gene selected from Dekkers et al. 2012*

[c]*Primers could not be designed on or over an intron-exon border*
