# Supplementary material for: GWA Mapping of Anthocyanin Accumulation Reveals Balancing Selection of MYB90 in Arabidopsis thaliana
Source: PLoS One. 2015 Nov 20;10(11):e0143212. doi: 10.1371/journal.pone.0143212 (PMC4654576; doi:10.1371/journal.pone.0143212)
Supplement: S2 Table — (PDF) [file pone.0143212.s008.pdf]

chro-mo-some	position	-log(p)	Minor allele frequency	Effect size	Explained genetic variance	Explained phenotypic variance	gene	gene annotation
1	24756576	13.92	0.15	0.7	27.87	10.02	promoter AT1G66380	MYB domain protein 114 (MYB114)
1	24758488	11.11	0.13	0.67	22.68	8.15	promoter AT1G66380	MYB domain protein 114 (MYB114)
1	24759985	16.98	0.14	0.81	35.06	12.6	promoter AT1G66390	MYB domain protein 90 (MYB90)
1	24760088	16.09	0.13	0.8	33.5	12.04	promoter AT1G66390	MYB domain protein 90 (MYB90)
1	24765632	8.85	0.29	0.42	16.39	5.89	promoter AT1G66400	calmodulin like 23 (CML23)
1	24767033	19.92	0.13	0.91	42.03	15.1	promoter AT1G66400	calmodulin like 23 (CML23)
1	24769084	11.75	0.2	0.57	23.5	8.45	promoter AT1G66400	calmodulin like 23 (CML23)
1	24769177	18.42	0.14	0.82	37.28	13.4	promoter AT1G66400	calmodulin like 23 (CML23)
1	24769757	16.48	0.17	0.73	33.11	11.9	promoter AT1G66400	calmodulin like 23 (CML23)
1	24769770	16.5	0.17	0.73	33.11	11.9	promoter AT1G66400	calmodulin like 23 (CML23)
1	24770871	7.74	0.33	0.38	14.44	5.19	exon AT1G66400	calmodulin like 23 (CML23)
1	24771021	7.72	0.32	0.38	14.43	5.19	exon AT1G66400	calmodulin like 23 (CML23)
